# Sensory neuron–derived Na_V_1.7 contributes to dorsal horn neuron excitability

**DOI:** 10.1126/sciadv.aax4568

**Published:** 2020-02-19

**Authors:** Sascha R. A. Alles, Filipe Nascimento, Rafael Luján, Ana P. Luiz, Queensta Millet, M. Ali Bangash, Sonia Santana-Varela, Xuelong Zhou, James J. Cox, Andrei L. Okorokov, Marco Beato, Jing Zhao, John N. Wood

**Affiliations:** 1 Molecular Nociception Group, Wolfson Institute for Biomedical Research, University College London, London WC1E 6BT, UK.; 2 Department of Neuroscience, Physiology and Pharmacology, University College London, London WC1E 6BT, UK.; 3 Synaptic Structure Laboratory, Instituto de Investigación en Discapacidades Neurológicas (IDINE), Department Ciencias Médicas, Facultad de Medicina, Universidad Castilla-La Mancha, Campus Biosanitario, C/Almansa 14, 02008 Albacete, Spain.; 4 Department of Anesthesiology, The First Hospital of Nanjing Medical University, Nanjing, Jiangsu 210029, China.

## Abstract

Expression of the voltage-gated sodium channel Na_V_1.7 in sensory neurons is required for pain sensation. We examined the role of Na_V_1.7 in the dorsal horn of the spinal cord using an epitope-tagged Na_V_1.7 knock-in mouse. Immuno–electron microscopy showed the presence of Na_V_1.7 in dendrites of superficial dorsal horn neurons, despite the absence of mRNA. Rhizotomy of L5 afferent nerves lowered the levels of Na_V_1.7 in the dorsal horn. Peripheral nervous system–specific Na_V_1.7 null mutant mice showed central deficits, with lamina II dorsal horn tonic firing neurons more than halved and single spiking neurons more than doubled. Na_V_1.7 blocker PF05089771 diminished excitability in dorsal horn neurons but had no effect on Na_V_1.7 null mutant mice. These data demonstrate an unsuspected functional role of primary afferent neuron-generated Na_V_1.7 in dorsal horn neurons and an expression pattern that would not be predicted by transcriptomic analysis.

## Teaser

An unsuspected functional role of primary afferent neuron-generated sodium channel Na_V_1.7 occurs in dorsal horn neurons.

## INTRODUCTION

The problem of pain continues to grow, and new analgesic approaches are urgently required (*1*). Human genetic studies have identified a number of potential analgesic targets, including neurotrophins, transcription factors, and ion channels (*2*–*4*). The sodium channel Na_V_1.7 expressed in sensory neurons is required for pain perception in mice and humans (*5*). Na_V_1.7 gain-of-function mutations result in ongoing pain in humans, while rare recessive loss-of-function mutants are pain free but otherwise normal apart from an inability to smell (*6*). Na_V_1.7 has been assumed to play an essential role in generating nociceptive spiking in sensory neurons. However, it has other roles in pain pathways. In mice, deletion of Na_V_1.7 in sensory neurons leads to enhanced expression of opioid peptides and potentiated opioid receptor activity (*7*, *8*). Much of the analgesia associated with loss of function of Na_V_1.7 in mice and humans can be reversed with the opioid antagonist naloxone, while Na_V_1.7 antagonist action is greatly potentiated by low-dose opioids or enkephalinase blockers (*9*, *10*). Na_V_1.7 has also an unusual role as an integrator of synaptic input in the hypothalamus (*11*).

Anosmia associated with loss of Na_V_1.7 in olfactory neurons has been shown to result from lack of glutamate release (*6*). In somatosensory neurons, there is also a loss of substance P release from Na_V_1.7 null mutant sensory neurons, suggesting a regulatory role for Na_V_1.7 in the control of neurotransmitter release (*12*). To examine this mechanism, we generated an epitope-tagged Na_V_1.7 knock-in mouse that shows entirely normal pain behavior. We used this mouse first to identify Na_V_1.7 interacting proteins using immunoprecipitation and mass spectrometry (*13*) and then to examine the expression of Na_V_1.7 in the central terminals of sensory neurons using both immunocytochemistry (ICC) and immuno–electron microscopy (immuno-EM).

## RESULTS

### Na_V_1.7 is expressed in primary afferent postsynaptic terminals in the dorsal horn

**Fig. 1:**
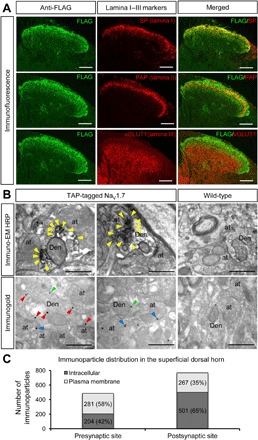
The distribution of Na_V_1.7 in the dorsal horn. (**A**) The distribution of TAP-tagged Na_V_1.7 in the dorsal horn was detected using immunofluorescence with anti-FLAG antibody (left). The cross sections of spinal cord (lumbar 5) were costained with markers of superficial dorsal horn such as substance P (lamina I), PAP (lamina II), and vGLUT1 (lamina III onwards) antibodies (middle). The right panels show the merged left to middle panels. The results show that TAP-tagged Na_V_1.7 mainly expresses in laminae I and II and in the part of lamina III. Scale bars, 100 μm. (**B**) Subcellular localization of TAP-tagged Na_V_1.7 in the dorsal horn of the spinal cord was identified with immuno-EM techniques. Electron micrographs showing immunolabeling for FLAG in the dorsal horn of the spinal cord were detected using the pre-embedding immunoperoxidase (top) and immunogold techniques (bottom). Using the pre-embedding immunoperoxidase method, peroxidase reaction end-product for FLAG in the TAP-tagged Na_V_1.7 mice was detected filling dendrites (Den) and dendritic spines of spinal cord neurons, as well as at presynaptic sites filling axon terminals (at). In the WT mice (top right), no peroxidase reaction end-product for FLAG was detected in the spinal cord. Using the pre-embedding immunogold method, immunoparticles for FLAG present in the TAP-tagged Na_V_1.7 were mainly detected at intracellular sites (arrowheads in red), as well as along the plasma membrane (arrowheads in green) in dendritic shafts (Den) of spinal cord neurons. In addition, immunoparticles for FLAG were observed along the extrasynaptic plasma membrane (arrowheads in blue) of axon terminals (at). In the WT mice (bottom right), a very low density of immunoparticles for FLAG, similar to background levels, was observed attached to mitochondria in the spinal cord. Scale bars, 500 nm. HRP, horseradish peroxidase. (**C**) The number of immunoparticles in the superficial dorsal horn of TAP-tagged Na_V_1.7 mice was counted. Of 1253 immunoparticles, 768 particles were located at postsynaptic sites (61%) and 485 particles were located at presynaptic sites (39%). Along the 768 postsynaptic particles, 501 particles were located at intracellular sites (65%), and 267 particles were located along the plasma membrane (35%). Along the 485 presynaptic particles, 204 particles were located at intracellular sites (42%), and 281 particles were located along the plasma membrane (58%).

Immunohistochemical studies showed that the Tandem Affinity Purification (TAP)–tagged Na_V_1.7 is expressed in laminae I and II and part of lamina III in the spinal cord on the basis of coexpression with substance P, prostatic acid phosphatase (PAP), and vesicular glutamate transporter 1 (vGLUT1) (Fig. 1A). These data are consistent with earlier studies that showed the coexpression of synaptophysin and Na_V_1.7 in lamina II of rat spinal cord (*14*). However, unexpectedly, immuno-EM showed that the TAP-tagged Na_V_1.7 is present not only in presynaptic sites of central terminals of peripheral sensory neurons but also in postsynaptic sites in dendrites in spinal cord neurons, as defined by ultrastructural criteria (Fig. 1B) (*15*, *16*). Quantitation of immunoreactive particles showed an association of 60% of immunoreactive Na_V_1.7 with postsynaptic sites, where a third of immunoparticles were present on the membranes of dendritic shafts (Fig. 1C). Further, immunofluorescence confocal microscopy was consistent with the finding that that the TAP-tagged Na_V_1.7 is expressed not only in the presynaptic terminals of sensory afferents but also in the postsynaptic terminals of interneurons in lamina II of the dorsal horn of the spinal cord (fig. S1).

### Sensory neurons are the source of Na_V_1.7 in dorsal horn neurons

**Fig. 2:**
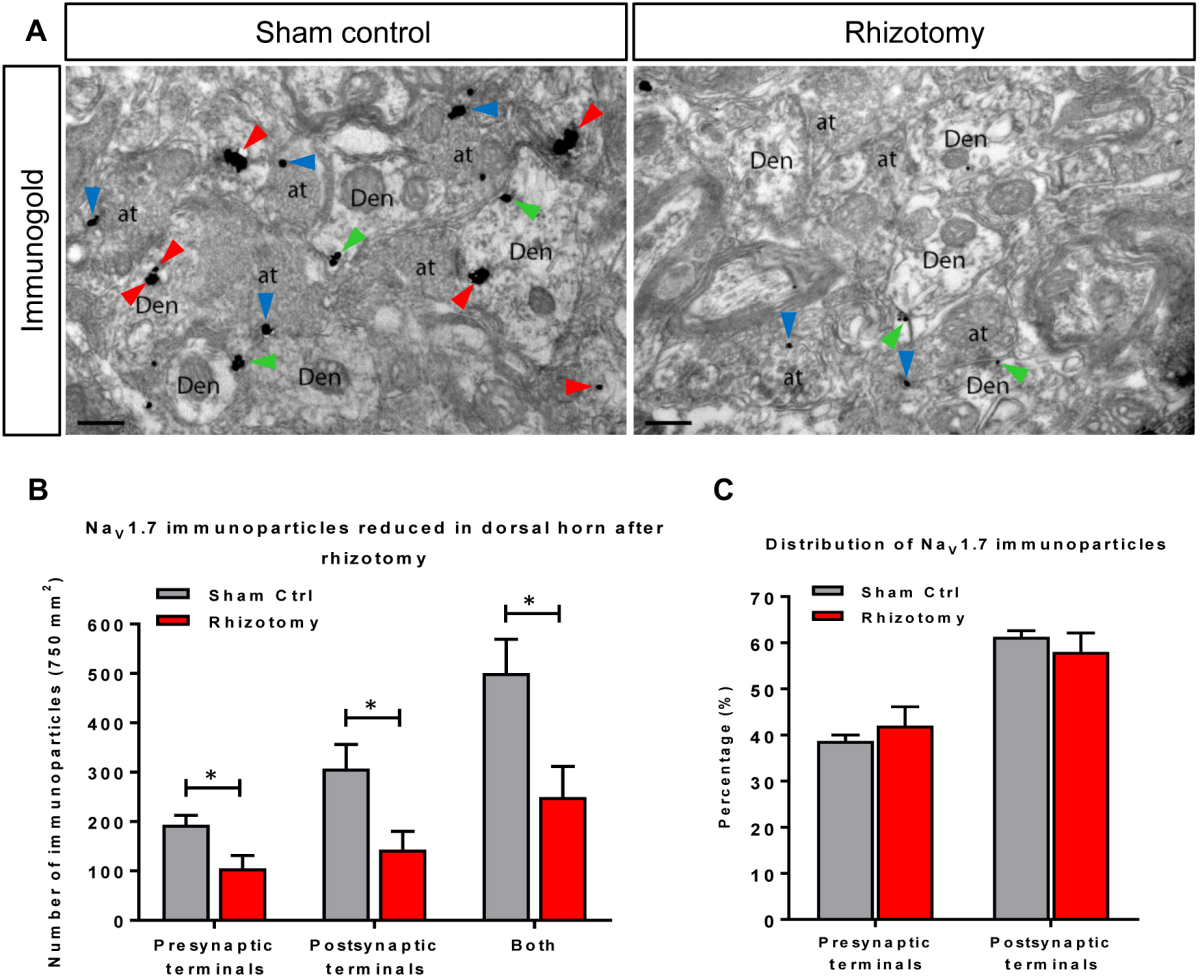
TAP-tagged Na_V_1.7 is down-regulated in the spinal cord after dorsal rhizotomy. Four weeks after dorsal rhizotomy on the right L5, the spinal cords of TAP-tagged Na_V_1.7 mice were extracted, and immuno-EM was performed with anti-FLAG antibody and pre-embedding immunogold techniques on the sections adjacent to L5. (**A**) Representative immuno-EM images from sham control (Sham Ctrl, left) and rhizotomy (right). Immunoparticles for FLAG were detected at intracellular sites (arrowheads in red), plasma membrane (arrowheads in green) in dendritic shafts (Den) of spinal cord neurons, and along the extrasynaptic plasma membrane (arrowheads in blue) of axon terminals (at). Scale bars, 200 nm. (**B**) The total numbers of immunoparticles from sham control samples (in gray) and rhizotomy (in red) indicate that the expression of TAP-tagged Na_V_1.7 is down-regulated by about 50% in both presynaptic and postsynaptic terminals after 4 weeks of rhizotomy. (**C**) Distribution (%) of immunoparticles in both presynaptic and postsynaptic terminals in the rhizotomy model. The numbers of immunoparticles were counted from both presynaptic and postsynaptic terminals. The result shows that there are no significant relative changes of distribution in the rhizotomy model. *P < 0.05, Student’s *t* test.

Does the dorsal horn neuron immunoreactive Na_V_1.7 originate from spinal cord mRNA transcripts? RNA sequencing studies of isolated neurons have identified some dorsal horn neurons that express Na_V_1.7 mRNA (*17*). Using the sensitive technique of RNAscope, the expression of Na_V_1.7 mRNA in vivo in a subset of motor neurons is apparent, but very few transcripts can be detected in dorsal horn cells (fig. S2) (*18*). We tested the origin of immunoreactive Na_V_1.7 in dorsal horn neurons using rhizotomy of L5 afferent spinal nerves followed by examination of TAP-tagged Na_V_1.7 by immuno-EM 4 weeks later (Fig. 2). The result shows that the number of immunoparticles in the region of rhizotomized spinal cord is significantly lower compared to L5 sham controls (Fig. 2, A and B). The total number of immunoparticles was reduced by about half in both presynaptic (axons) and postsynaptic terminals (dendrites) 4 weeks after rhizotomy (Fig. 2B). Compared to controls, no obvious changes were found in the relative distribution of immunoparticles in dorsal horn of rhizotomized mice, where approximately 40% residual particles were found in presynaptic terminals and 60% in postsynaptic terminals in both sham control and rhizotomized mice (Fig. 2C). The residual immunoreactivity in rhizotomized dorsal horn neurons may represent degradation products or protein derived from ascending or descending primary afferents entering the cord through the adjacent nontransected roots. The lifetime of Na_V_1.7 protein has been suggested to be in the region of weeks on the basis of adult deletion of the *Scn9a* gene in mice (*19*). Together, our evidence is consistent with the view that afferent nerves are the source of the immunoreactive Na_V_1.7 in the dorsal horn.

### Electrophysiological properties of lamina II neurons are altered in primary afferent Na_V_1.7 knockout mice

**Fig. 3:**
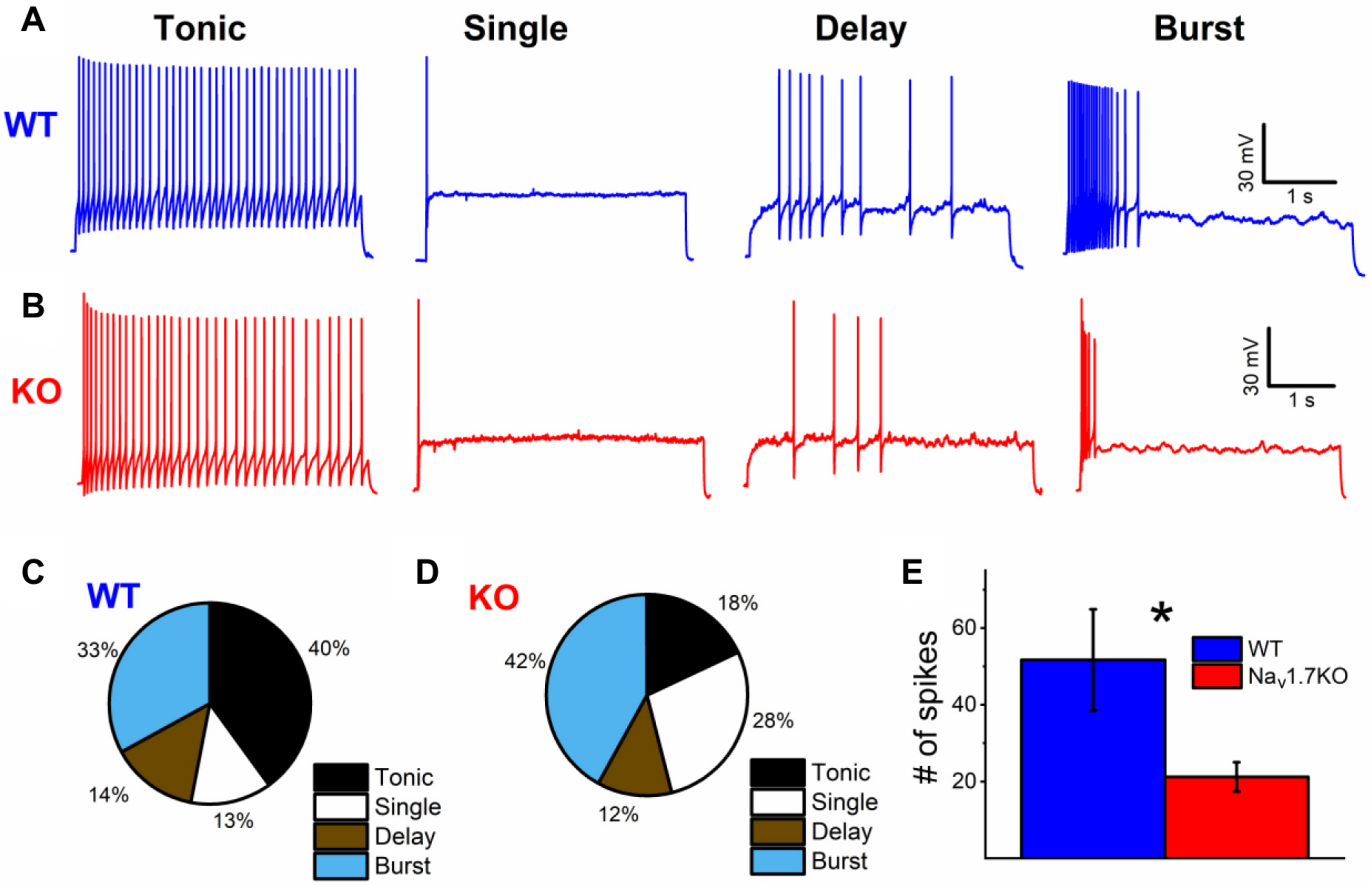
Electrophysiological properties of lamina II neurons are altered in sensory neuron–specific Na_V_1.7 KO mice. Representative traces of the four main firing patterns of lamina II neurons recorded from ex vivo spinal cord slices from (**A**) WT and (**B**) Na_V_1.7 KO mice. Current injections are not shown for clarity. Current injections for recordings shown were as follows: WT tonic = 80 pA, WT single = 200 pA, WT delay = 200 pA, WT burst = 60 pA, KO tonic = 30 pA, KO single = 100 pA, KO delay = 70 pA, and KO burst = 50 pA. Percentage of each neuronal subpopulation in lamina II from (**C**) WT (*n* = 83 neurons) and (**D**) Na_V_1.7 KO (*n* = 50 neurons) mice. (**E**) Maximum number of spikes elicited by current injection for each neuron compared between WT and Na_V_1.7 KO mice. The difference is statistically significant (*P = 0.02875, two-sample *t* test with Welch correction) with WT at 51.7 ± 13.2 spikes (*n* = 83 neurons) and Na_V_1.7 KO at 21.2 ± 3.8 spikes (*n* = 50 neurons).

To explore any possible functional relevance of channel transfer, we examined the electrophysiological properties of dorsal horn lamina II neurons from mice where Na_V_1.7 is deleted only in sensory neurons using Cre-recombinase driven by the advillin promoter. Lamina II neurons exhibit different firing properties following somatic current injections and can be classified according to their firing pattern as tonic, burst, single spike, or delayed firing (*20*). We investigated whether there were differences in the relative frequency of each type of neuron between wild-type (WT) and Dorsal Root Ganglion (DRG)–specific Na_V_1.7 knockout (KO) mice. All four types of firing pattern were observed in both WT and Na_V_1.7 KO animals, as shown in the representative traces of Fig. 3 (A and B). The prevalence of delay neurons was similar in WT (12 of 83) and Na_V_1.7 KO (6 of 50) mice, but the percentage of tonic neurons in Na_V_1.7 KO mice was less than half that in WT (Na_V_1.7 KO = 18% versus WT = 40%) mice. The reduction in the proportion of tonic neurons in Na_V_1.7 KO mice was compensated by the prevalence of single spike (Na_V_1.7 KO = 28% versus WT = 13%; Fig. 3, C and D) and, to a lesser extent, bursting cells (42% versus 33%; Fig. 2, C and D). The differences in neuronal populations in Na_V_1.7 KO compared to WT dorsal horn was statistically significant (*P* = 0.0018, χ^2^ test). Our data suggest that dorsal horn neurons from Na_V_1.7 peripheral neuron KO mice have reduced excitability. This was confirmed by the observation that the maximum number of spikes recorded during a 4-s maximal current pulse (sufficient to evoke firing at maximal frequency without spike inactivation) was reduced (*P* = 0.0288, two-sample *t* test with Welch correction; Fig. 3E), with WT producing 51.7 ± 13.2 spikes (*n* = 83 neurons) and Na_V_1.7 KO generating 21.2 ± 3.8 spikes (*n* = 50 neurons). We also demonstrated that there is a significant increase in the AP threshold in Na_V_1.7 KO mice compared to WT neurons (*P* = 0.0483, two-sample *t* test with Welch correction; fig. S3D). As these events occur in mice where Na_V_1.7 is deleted only in sensory neurons, the deficits in dorsal horn neurons must result from a lack of translocated Na_V_1.7.

### Na_V_1.7 blocker PF05089771 diminishes excitability in dorsal horn neurons

**Fig. 4:**
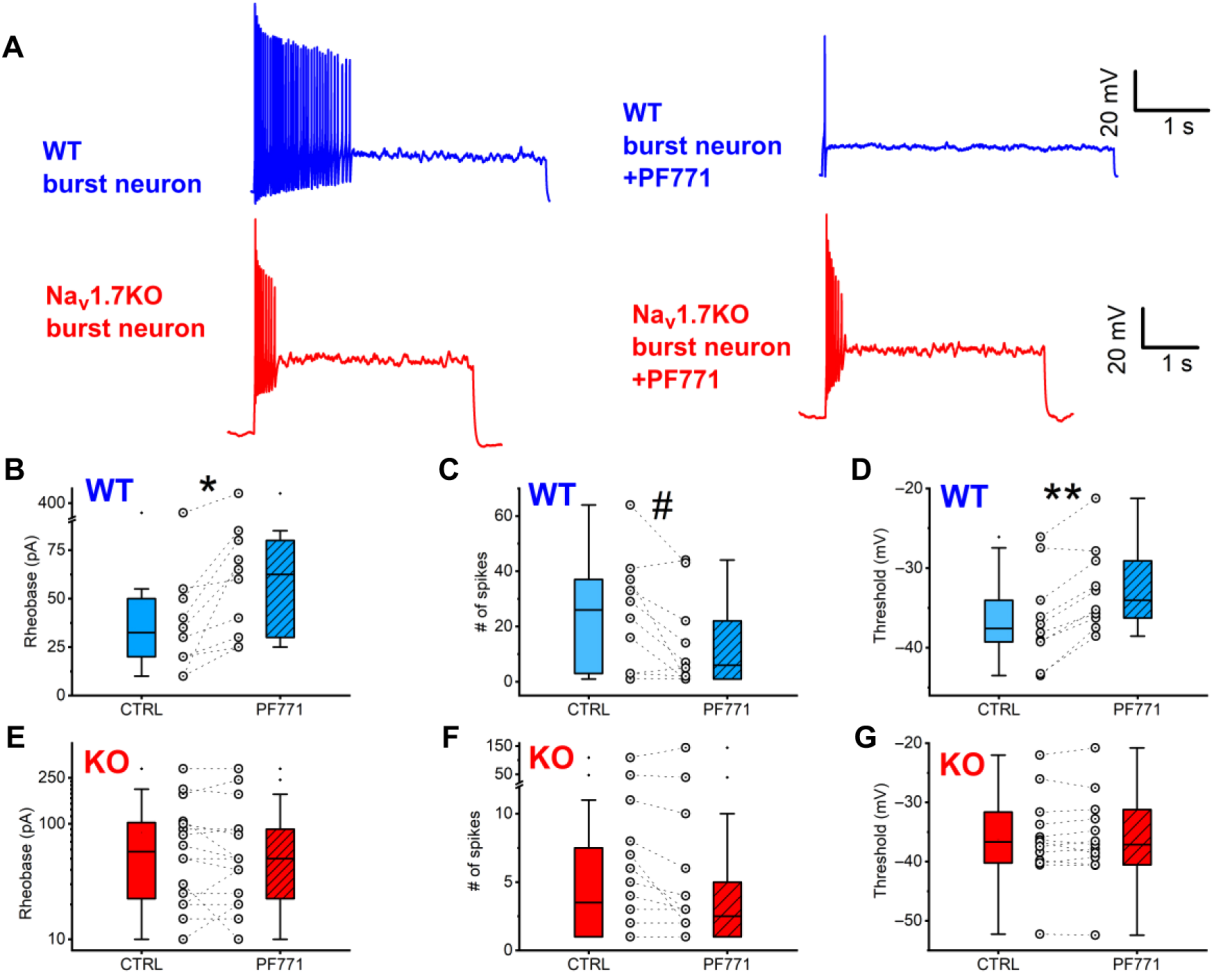
Effect of specific Na_V_1.7 blocker PF771 on lamina II neurons from WT and Na_V_1.7 KO mice. Two populations of WT neurons were identified: neurons that displayed an increase in rheobase and neurons that showed little or no change in rheobase in the presence of PF771 (see fig. S4). (**A**) Representative WT burst firing neuron displaying an effect of PF771 on firing. (**B**) Representative Na_V_1.7 KO burst firing neuron displaying no effect of PF771 on firing. Current injections for traces were as follows: WT burst = 30 pA and Na_V_1.7 KO burst = 50 pA. Current injections were identical before and after drug. (**B**) WT neurons were split on the basis of rheobase change in the presence of PF771. Paired WT neurons displayed an increase in rheobase with PF771 (hatched bar) versus control (open bar; *P = 0.00586, paired Wilcoxon signed rank test) (**C**) Maximum number of spikes (at the same current injection before and after drug) was significantly reduced with PF771 versus control (^#^*P* = 0.04383, paired Wilcoxon signed rank test) in WT neurons showing an increase in rheobase only. (**D**) Threshold was significantly increased with PF771 versus control (**P = 0.00178, paired Wilcoxon signed rank test) in WT neurons showing an increase in rheobase only. There were no major population differences in terms of response to PF771 identified in Na_V_1.7 KO mice dorsal horn neurons. There were no significant changes in (**E**) rheobase, (**F**) number of spikes, or (**G**) threshold of Na_V_1.7 KO dorsal horn neurons in the presence of PF771 versus control.

We extended this genetic analysis by recording from lamina II neurons in WT mice to determine their firing properties before and after application of PF05089771 (henceforth PF771), a selective blocker of Na_V_1.7 channels (*21*). Application of the blocker changed the firing pattern in a minority of cells (6 of 24; Fig. 4A, top). Three burst firing neurons became single spiking neurons, two tonic firing neurons became burst firing, and one tonic firing neuron became single spiking in the presence of PF771. In addition, in 10 of 24 neurons, the rheobase significantly increased following application of PF771 (median, 32.5 to 62.5 pA; *P* = 0.00586, paired Wilcoxon signed rank test; Fig. 4B). Consistently, in those cells in which PF771 increased rheobase, the median of the maximum number of spikes decreased from 26 to 6 spikes (*P* = 0.0438, paired Wilcoxon signed rank test). In contrast, in 14 of 24 cells in which rheobase was not significantly affected (median, 25 to 22.5 pA; *P* = 0.1; fig. S3B), the median number of spikes did not change significantly (median, 33.5 and 36.5; *P* = 0.358, paired Wilcoxon signed rank test; fig. S3C). In the neurons in which rheobase was increased, the voltage threshold was also differentially affected: In the groups of cells responding to PF771, the median voltage threshold was −37.6 mV in control and increased to −34 mV (*P* = 0.00805, paired Wilcoxon signed rank test), while it did not change for the nonresponding cells (median, −37.4 to −38 mV; *P* = 0.286; fig. S4).

Note that the block by PF771 at this concentration is only effective after channels have been inactivated by a prolonged depolarization (cells that exhibited a change in input resistance of 10% or more following this depolarization were discarded from further analysis; see Methods). Because any Na_V_1.7 channels located on primary afferents would not have been inactivated and would be insensitive to PF771, the effects observed are specific to postsynaptic Na_V_1.7 on dorsal horn neurons. These data confirm that functional Na_V_1.7 channels are expressed on the postsynaptic membrane of a subset of lamina II neurons, and their activation contributes to neuronal excitability.

To confirm the specificity of the action of PF771, we repeated the same experiments with Na_V_1.7 KO mice, measuring the electrophysiological properties of *n* = 16 lamina II neurons. In Na_V_1.7 KO mice, there was no significant change in rheobase (median, 57.5 to 50 pA; *P* = 0.504), maximum number of spikes (median, 3.5 to 2.5; *P* = 0.181), or threshold (−36.7 to −37.1 mV; *P* = 0.514) in the presence of PF771 (Fig. 4, E to G). This indicates that KO of Na_V_1.7 in presynaptic afferent sensory neurons results in a functional deficit postsynaptically in the superficial dorsal horn.

## DISCUSSION

Our functional studies demonstrate that the excitability, threshold, and firing pattern of a subset of dorsal horn neurons can be ascribed to the presence of primary afferent sensory neuron–derived Na_V_1.7. These findings have significance for analgesic drug development and help to explain the failure of peripherally restricted Na_V_1.7 antagonists to be effective analgesics (*9*). Na_V_1.7 loss, apart from effects on sensory neuron excitability, increases endogenous opioid drive, inhibits neurotransmitter release, and, as we have shown here, diminishes dorsal horn excitability. The relative importance of these regulatory steps in Na_V_1.7 loss-of-function analgesia is uncertain. Nonetheless, our findings demonstrate intercellular transport of Na_V_1.7, a functional role for the channel in recipient cells, and a pattern of Na_V_1.7 expression that differs from that predicted by transcriptomic studies. Na_V_1.7 antagonists have yet to produce as profound analgesia as loss of the encoding gene itself, probably because complete channel block seems to be required to induce opioid peptide expression (*7*). These results support the view that targeted ablation of *Scn9a* in sensory neurons may be a more productive approach to analgesia than the development of small-molecule antagonists of Na_V_1.7.

What is the mechanism of trans-neuronal transfer of Na_V_1.7? Evidence for protein transfer between cells has been obtained in the immune system (trogocytosis) (*22*), with transcription factors (*23*), through trans-synaptic transfer and via exosome release (*24*). The trans-neuronal transfer of synuclein has been linked to neurodegenerative disease (*25*). Recent studies also demonstrate trans-synaptic transfer of RNA via the activity-dependent immediate early retrotransposon capsid–like ARC protein that is linked to synaptic plasticity, memory formation, and neuropsychiatric disease (*26*). It seems that circuitry within the brain involves not only electrical signaling but also activity-dependent transfer of RNA and proteins. This is the first example of peripherally encoded proteins exerting effects on central nervous system function. However, the mechanism of Na_V_1.7 transfer remains to be established.

## METHODS

### Animals

Both female and male mice aged 4 to 8 weeks were kept on a 12-hour light/dark cycle and provided with food and water ad libitum. Conditional Na_V_1.7 KO mice were generated by crossing floxed (SCN9A) Na_V_1.7 mice with Advillin-Cre mice (*27*). Na_V_1.7^TAP^ knock-in mice were generated as described previously (*13*). All experiments were performed with approval from the United Kingdom Home Office according to guidelines set by personal and project licenses, as well as guidelines of the Committee for Research and Ethical Issues of IASP (International Association for the Study of Pain).

### Immunocytochemical studies

The perfusion and staining were performed as previously described (*28*). Briefly, after deep anesthesia with pentobarbitone sodium solution (80 mg/kg; Pentoject, 57-33-0, Animal-care) by intraperitoneal injection (e.g., 20 μl/25 g of mouse), animals were transcardially perfused with 10 ml of heparinized saline [0.9% (w/v) NaCl; heparin (10 U/ml), Wockhardt UK Ltd.], followed by 25 ml of freshly prepared 4% (w/v) paraformaldehyde in 0.1 M phosphate buffer (PB; pH 7.4) (containing 15% saturated picric acid and 0.1% glutaraldehyde for immuno-EM). After perfusion, the spinal cords were removed, postfixed in the same fixative solution at 4°C overnight, and then cryoprotected in 30% (w/v) sucrose containing 0.02% sodium azide in 0.1 M PB overnight. Subsequently, the sections were cut at a thickness of 60 μm on a vibratome (VT1000S, Leica), then incubated in 50% ethanol for 30 min, blocked in a blocking buffer [1× phosphate-buffered saline (PBS) containing 0.3% Triton X-100 containing 5% donkey serum] at room temperature for 1 hour, and then incubated in primary antibodies diluted in the blocking buffer at 4°C overnight. After three washes in PBS, the sections were incubated in the secondary antibodies at room temperature in the dark for 2 hours. Later, the sections were rinsed three times in PBS and then placed onto SuperFrost Plus (Ref J1800AMNZ, Thermo Fisher Scientific) slides, mounted in an Antifade Mounting Medium (H-1000, Vector Laboratories), covered with coverslip, and sealed with nail varnish. Last, the sections were scanned with a Zeiss LSM 710 204 confocal microscope. The primary antibodies are anti-FLAG (1:100; F1804, Sigma), anti–substance P (1:200; OBT06435, Oxford Biotec), anti-PAP (1:1000; AAF23171, Aves Lab Inc.), anti-vGLUT1 (1:5000; AB5905, Chemicon), anti-Homer (1:1000; Frontier Institute, AB_2631104), and anti-vGLUT2 (1:5000; ab2251, Millipore). The secondary antibodies are donkey anti-mouse A488 (1:1000; 715-545-150, Jackson ImmunoResearch), donkey anti-chicken (1:1000; 703-025-155, Jackson ImmunoResearch), donkey anti-rat A647 (1:1000; 712-605-153, Jackson ImmunoResearch), donkey anti–guinea pig Pacific Blue (1:1000; 706-475-148, Jackson ImmunoResearch), and donkey anti-goat Rhodamine Red (1:1000; 705-025-003, Jackson ImmunoResearch).

### Immunohistochemistry for electron microscopy

Immunohistochemical reactions for electron microscopy were carried out using the pre-embedding immunoperoxidase and immunogold methods described previously (*15*). Briefly, 10-week-old TAP-tagged Na_V_1.7 mice (*n* = 3) and WT littermate control mice (*n* = 3) were used for immuno-EM. After being deeply anesthetized, the cross sections from lumbar 4 to 5 of spinal cords were prepared as above (immunofluorescence). Subsequently, the free-floating sections were blocked and incubated with a monoclonal anti-FLAG (3.5 μg/ml; F1804, Sigma) antibody in blocking buffer [tris-buffered saline (TBS) containing 1% (v/v) normal goat serum (NGS)]. They were incubated in biotinylated goat anti-mouse immunoglobulin G (IgG; Vector Laboratories) or in goat anti-mouse IgG coupled to 1.4 nm of gold (Nanoprobes Inc., Stony Brook, NY, USA) diluted in TBS containing 1% NGS. For immunoperoxidase, the sections were then transferred to an avidin-biotinylated peroxidase mixture (ABC kit, Vector Laboratories) diluted as 1:100 for 2 hours at room temperature. Peroxidase enzyme activity was revealed by using a 3,3′-diaminobenzidine tetrahydrochloride solution (0.05% in TB, pH 7.4), to which 0.01% H_2_O_2_ was added. For immunogold, the sections were postfixed in 1% (v/v) glutaraldehyde and washed in double-distilled water, followed by silver enhancement of the gold particles with an HQ Silver kit (Nanoprobes Inc.). All sections were then treated with osmium tetraoxide (1% in 0.1 M PB), block-stained with uranyl acetate, dehydrated in graded series of ethanol, and flat-embedded on glass slides in Durcupan (Fluka) resin. Regions of interest were cut at 70 to 90 nm on an ultramicrotome (Reichert Ultracut E, Leica, Austria) and collected on single-slot pioloform-coated copper grids. Staining was performed on drops of 1% aqueous uranyl acetate, followed by Reynolds’s lead citrate. Ultrastructural analyses were performed in a JEOL JEM-1010 electron microscope.

### Dorsal rhizotomy and immuno-EM

The procedure of dorsal rhizotomy in the region of lumbar dorsal root ganglion 5 (L5) was performed as described (*29*). Briefly, 8-week-old TAP-tagged Na_V_1.7 mice were anesthetized with 2 to 3% isoflurane in oxygen (0.5 liter/min), and then a laminectomy on L5 of spinal cord was followed by unilateral transection of the right L5 dorsal roots (Rhizotomy). For the sham controls (Sham Ctrl), the same procedures were performed as rhizotomy except transection. Four weeks after the surgery, the mice were perfused with 4% paraformaldehyde in PBS containing 0.1% glutaraldehyde (Sigma, G5882). After perfusion, the spinal cords were removed and postfixed in the same fixative at 4°C overnight. Then, the tissues were placed in 30% sucrose in 1× PBS at 4°C overnight. The spinal cords were sectioned, and immuno-EM was performed as described in the main text. For counting the number of immunoparticles in laminae I, II, and III of right L5 spinal cords, 50 electronic microscope images were randomly taken from each sample. The surface area of each image is 15 μm^2^. Therefore, the total number of each sample will represent immunoparticles in the surface of 750 μm^2^.

### Spinal cord preparation

Spinal cord preparations were obtained from male or female mice, between 30 and 60 days old, from either WT C57Bl/6 or conditional Na_V_1.7 KO (Na_V_1.7 KO). Animals were anesthetized via intraperitoneal injection of a ketamine/xylazine mix (80 and 10 mg/kg, respectively) and decapitated. The spinal cord was dissected in ice-cold artificial cerebrospinal fluid (aCSF) of the following composition: 113 mM NaCl, 3 mM KCl, 25 mM NaHCO_3_, 1 mM NaH_2_PO_4_, 2 mM CaCl_2_, 2 mM MgCl_2_, and 11 mM d-glucose, and the same solution was used for subsequent electrophysiological recordings. Once dissected free from the vertebral column, the spinal cord was carefully cleaned from connective tissues, and dorsal roots were cut at approximately 2 mm in length. The spinal cord was then glued to an agar block and glued to the slicing chamber of an HM 650V vibratome (Microm, Thermo Fisher Scientific, UK). The solution used for slicing contained 130 mM k-gluconate, 15 mM KCl, 0.05 mM EGTA, 20 mM Hepes, 25 mM d-glucose, 3 mM kynurenic acid, 2 mM Na-pyruvate, 3 mM myo-inositol, 1 mM Na-l-ascorbate, and pH 7.4 with NaOH (*30*). Slices were incubated for 40 min at 35° and then allowed to equilibrate at room temperature for a further 30 min before starting the recordings.

### Electrophysiology

Current and voltage clamp recordings were performed using either a Molecular Devices Multiclamp 700B amplifier (Scientifica, UK) or an ELC-03X amplifier (NPI electronic, Germany). Signals were filtered at 5 KHz, acquired at 50 KHz using a Molecular Devices 1440A A/D converter (Scientifica, UK), and recorded using Clampex 10 software (Molecular Devices, Scientifica, UK). Electrodes were pulled with a Flaming-Brown puller (P1000, Sutter Instruments, USA) from borosilicate thick glass (GC150F, Harvard Apparatus, UK). The resistance of the electrodes, following fire polishing of the tip, ranged between 3 and 5 megohms. Bridge balance was applied to all recordings. Intracellular solution contained 125 mM k-gluconate, 6 mM KCl, 10 mM Hepes, 0.1 mM EGTA, 2 mM Mg–adenosine triphosphate (ATP), pH 7.3 with KOH, and osmolarity of 290 to 310 mOsm. Cells were included for further analysis if their input resistance throughout the duration of the experiment was stable (within 10% of its initial value) and if their spikes (evoked by a current step) peaked beyond 5 mV.

Cells were targeted in the inner and outer lamina II and visualized using an Eclipse E600FN Nikon microscope (Nikon, Japan) equipped with infrared differential interference contrast connected to a digital camera (Nikon, DS-Qi1Mc). In most experiments, a dorsal root was stimulated via a section electrode connected to an isolated current stimulator (DS3, Digitimer, UK). The stimulation intensity was fixed at 5× threshold for evoking the low threshold response in the recorded cell (typically 50 to 100 μA). Cells were held in current clamp mode, and their resistance and capacitance were measured from the voltage response to a brief (20 ms) current step (10 to 20 pA). Rheobase was defined as the minimum current necessary to elicit a spike. Cells were classified into tonic (firing continuously throughout a current step), single spike (firing only once at the beginning of the current step), delayed (firing during the course of the current step), and bursting (firing more than one spike at the start of the current step).

PF771 was bath-applied through perfusion at a concentration of 100 nM. It has been shown that at our chosen concentration of PF771 has no blocking effect on Na_V_1.7 unless the channels are inactivated (*21*). In our experimental conditions, the response to current injection was never affected by the presence of the blocker. The drug effects were observed only after Na_V_1.7 channels were inactivated by holding the cell at depolarized potential (0 mV) for 30 s. Cells that exhibited a change in input resistance of 10% or more following this depolarization were discarded from further analysis. Cells were defined as “responsive” to the channel blocker if their rheobase increased by at least 10% in the presence of PF771. All drugs were obtained from Sigma-Aldrich unless stated otherwise.

### Data analysis and statistics

For electrophysiological data, the spike kinetic parameters (spike width, height, threshold, and maximum rate of rise and descent) were measured using custom-written software [Bayesian Quantal Analysis suite, freely downloadable at http://sourceforge.net/projects/pyclamp/ (*31*)]. All other statistical and data analyses were performed with Origin Lab 2018, GraphPad Prism 7.0, Microsoft Excel, or Zeiss Zen Blue microscopy software. Values are expressed as the means ± SEM. The statistical significance of the data was analyzed using Student’s *t* test. Significant differences between two groups were examined statistically as indicated (*P < 0.05, **P < 0.01).

## Supplementary Material

http://advances.sciencemag.org/cgi/content/full/6/8/eaax4568/DC1

Download PDF

Sensory neuron–derived NaV1.7 contributes to dorsal horn neuron excitability

## SUPPLEMENTARY MATERIALS

Supplementary material for this article is available at http://advances.sciencemag.org/cgi/content/full/6/8/eaax4568/DC1 Supplementary Materials and Methods Fig. S1. The distribution and colocalization of Na_V_1.7 in the dorsal horn. Fig. S2. Levels and distribution of *Scn9a* transcript in mouse spinal cord. Fig. S3. Intrinsic properties of WT and Na_V_1.7 KO superficial dorsal horn neurons. Fig. S4. “PF771-unresponsive” WT superficial dorsal horn neurons. References (*32*, *33*)

## Appendix

View/request a protocol for this paper from *Bio-protocol*.
