# Supplementary material for: Sensory neuron–derived NaV1.7 contributes to dorsal horn neuron excitability
Source: Sci Adv. 2020 Feb 19;6(8):eaax4568. doi: 10.1126/sciadv.aax4568 (PMC7030926; doi:10.1126/sciadv.aax4568)
Supplement: http://advances.sciencemag.org/cgi/content/full/6/8/eaax4568/DC1 [file supp_6_8_eaax4568__index.html]

Science Advances | Science AdvancesAAASSearchScience AdvancesMenu

## Supplementary Materials

**This PDF file includes:**

- Supplementary Materials and Methods
- Fig. S1. The distribution and colocalization of NaV1.7 in the dorsal horn.
- Fig. S2. Levels and distribution of *Scn9a* transcript in mouse spinal cord.
- Fig. S3. Intrinsic properties of WT and NaV1.7 KO superficial dorsal horn neurons.
- Fig. S4. “PF771-unresponsive” WT superficial dorsal horn neurons.
- References (*32*, *33*)

Download PDF

**Files in this Data Supplement:**

- Adobe PDF - aax4568\_SM.pdf
