# Supplementary material for: Sensory neuron–derived NaV1.7 contributes to dorsal horn neuron excitability
Source: Sci Adv. 2020 Feb 19;6(8):eaax4568. doi: 10.1126/sciadv.aax4568 (PMC7030926; doi:10.1126/sciadv.aax4568)
Supplement: Download PDF [file aax4568_SM.pdf]

Science Advances
AAAS

advances.sciencemag.org/cgi/content/full/6/8/eaax4568/DC1

Supplementary Materials for

Sensory neuron–derived Na_V1.7 contributes to dorsal horn neuron excitability

Sascha R. A. Alles, Filipe Nascimento, Rafael Luján, Ana P. Luiz, Queensta Millet, M. Ali Bangash, Sonia Santana-Varela, Xuelong Zhou, James J. Cox, Andrei L. Okorokov, Marco Beato*, Jing Zhao*, John N. Wood*

*Corresponding author. Email: m.beato@ucl.ac.uk (M.B.); jing02.zhao@ucl.ac.uk (J.Z.); j.wood@ucl.ac.uk (J.N.W.)

Published 19 February 2020, *Sci. Adv.* **6**, eaax4568 (2020)
DOI: 10.1126/sciadv.aax4568

This PDF file includes:

Supplementary Materials and Methods
Fig. S1. The distribution and colocalization of Na_V1.7 in the dorsal horn.
Fig. S2. Levels and distribution of *Scn9a* transcript in mouse spinal cord.
Fig. S3. Intrinsic properties of WT and Na_V1.7 KO superficial dorsal horn neurons.
Fig. S4. "PF771-unresponsive" WT superficial dorsal horn neurons.
References (*32*, *33*)

Supplementary Materials and Methods

In situ Hybridization (ISH)

Animals were perfused with 4% paraformaldehyde in phosphate-buffered saline (PBS, pH 7.4). After perfusion, lumbar spinal cord segments (L3-L5) were removed and postfixed in the same fixative for 2 hours at 4°C.For *in situ* hybridization, lumbar spinal cord segments were dissected and post-fixed for 2 hours at 4°C. The spinal cords were cryo-sectioned to 10-11 um, thaw-mounted onto Superfrost Plus (Fisher Scientific) slides, allowed to dry for 30 minutes at RT, and then stored at −80 °C.

In situ hybridization was performed using the RNAscope system (Advanced Cell Diagnostics) (*32*) following protocol for fresh-frozen samples with 1h postfixing with 4% PFA in PBS at 4°C and stepwise dehydration with 50%, 70% and 100% Ethanol. Tissue pre-treatment consisted of hydrogen peroxide and Protease IV (10 and 20 min respectively) at RT. Following pre-treatment, probe hybridization and detection with the Multiplex Fluorescence Kit v2 were performed according to the manufacturer's protocol. Probes included NeuN (RbFox3 #313311) and Scn9a (#313341). RNA localisation was detected with Opal 520 (green) and Opal 650 (far-red) fluorochrome dyes (Perkin Elmer) compared to DAPI staining (nuclei) or TS-coumarin (TS405, Perkin Elmer) used for NeuN. Following ISH slides were mounted using Prolong Gold (ThermoFisher Scientific #P36930). Fluorescence was detected using a Zeiss LSM 880 Airyscan microscope. Images were taken at 10x and 20x magnification with 4x averaging (typically stitched 16-20 tiles), ayriscan processed and exported as 16-bit uncompressed tiff files for further basic editing in Adobe Lightroom v7 (Adobe) on colour calibrated iMac retina monitor. Final images were exported as jpeg files with 7,200 pix on longest side.

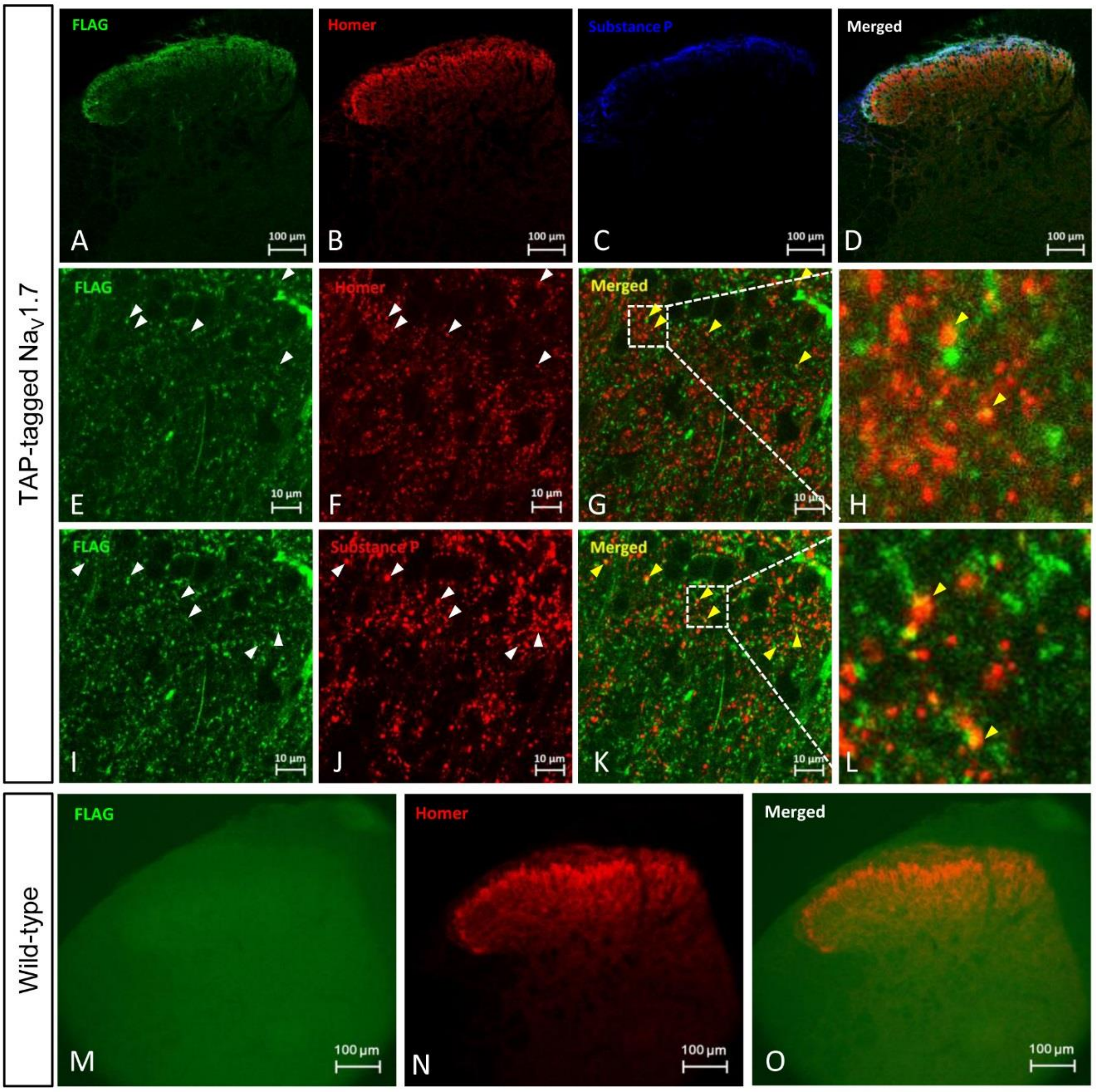

Fig. S1. The distribution and colocalization of $Na_V1.7$ in the dorsal horn. The distribution and co-localisation of $Na_V1.7$ in the dorsal horn of TAP-tagged $Na_V1.7$ mice were also detected using immunofluorescence and confocal microscopy with both presynaptic marker Substance P (SP) and postsynaptic marker Homer, especially for excitatory synapses of interneurons in dorsal horn. (**A**-**D**) Low magnification (20x) views show that the immunoreactivity of FLAG (TAP-tagged $Na_V1.7$) is present in Lamina I, II and part of III compared to Homer mainly in Lamina II and III, and Substance P in Lamina I. Higher magnification (63x) views of parts of Lamina II (**E**-**H**) and Lamina I (**I**-**L**) show that the immunostainings are in the form of small puncta that are scattered throughout the neuropil (E-G and I-K). The yellow dots pointed by headarrows (in yellow) in panel G and

H, and in panel K and L indicate the co-localisation of Na_V1.7-Homer and Na_V1.7-SP in superficial Lamina II and I, respectively. The cross sections from dorsal horn of wild-type littermate control mice were stained with anti-FLAG antibody and anti-homer antibody (**M-O**) as controls. Scale bars = 100 μm (A-D and M-O), and 10 μm (E-G and I-K).

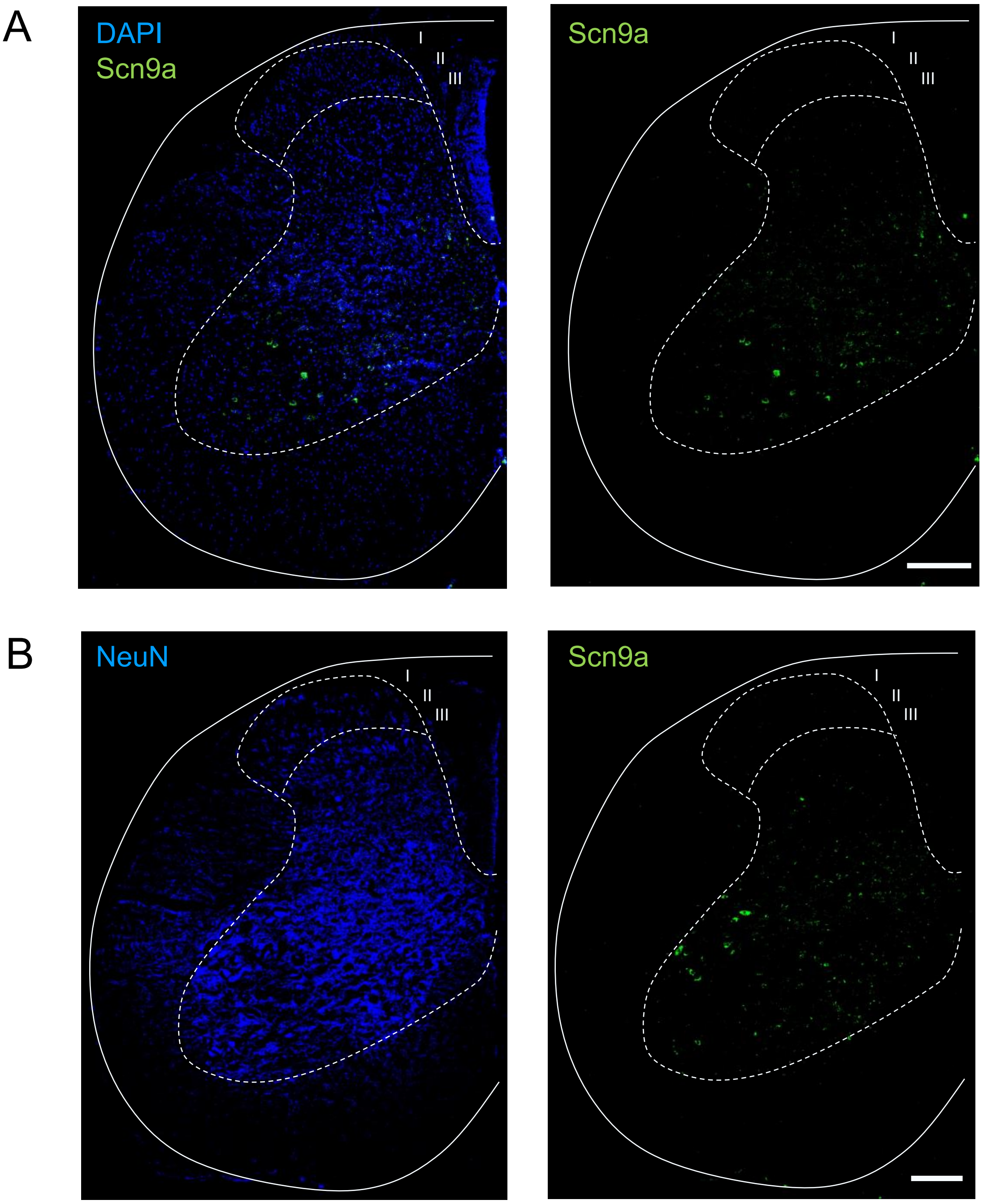

Fig. S2. Levels and distribution of *Scn9a* transcript in mouse spinal cord. 10-11 µM - thick fresh-frozen cuts of L5 section of spinal cord were fixed with 4% PFA and analysed by RNAscope Multiplex Fluorescent reagent assay v2 (ACD) using probes for NeuN (RbFox3 Cat# 313311) and Scn9a (Cat#313341). (**A**) RNA localisation of Scn9a (green, Opal520) compared to DAPI staining. (**B**) RNA localisation of Scn9a (green, Opal520) compared to that of NeuN (blue, TS405) RNAscope signals. Scale bar = 200 µm.

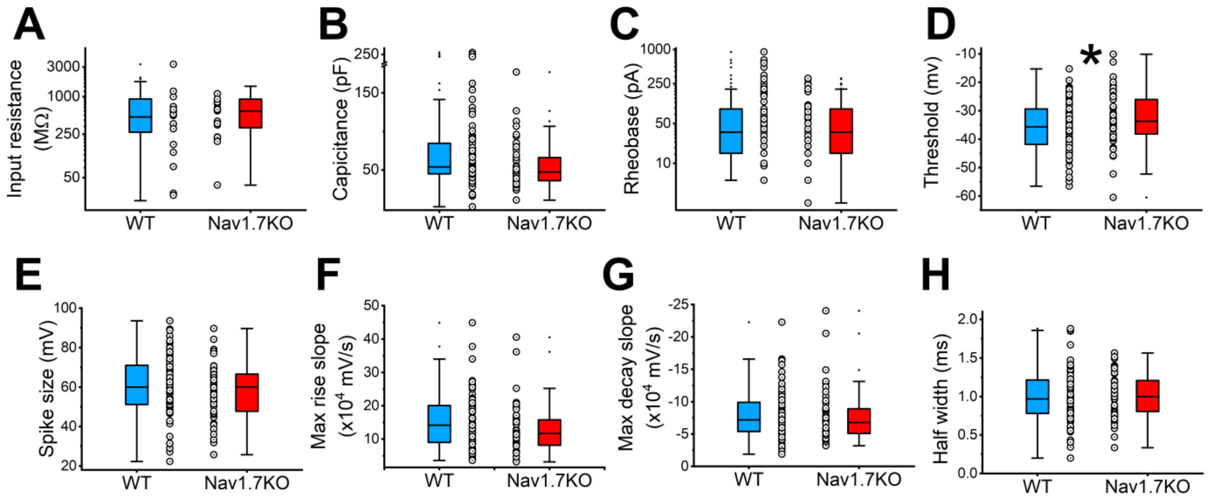

Fig. S3. Intrinsic properties of WT and Na$_V$1.7 KO superficial dorsal horn neurons. (**A**) Input resistance (in MΩ) (**B**) capacitance (pF) (**C**) rheobase (pA) (**D**) threshold (mV)**. E**. spike size (mV) (**F**) max rise slope (x10^4 mV/ms) and (**G**) descent (x10^4 ms) and (**H**) AP half-width (ms) of WT (light blue, n = 83 neurons) and Na$_V$1.7 KO dorsal horn neurons (red, n = 50 neurons). Threshold was the only property that was significantly increased in Na$_V$1.7 KO dorsal horn neurons compared to WT dorsal horn neurons (*p = 0.04834, two-sample t-test with Welch correction). Interestingly, it has been shown that there is a decrease in AP threshold in nociceptors derived from chronic pain patients with inherited erythromelalgia (IEM) harbouring a gain-of-function mutation in the Na$_V$1.7 channel, which agrees with our data (*33*). Input resistance, capacitance, rheobase, spike size, max rise slope, descent and half width were not significantly changed (p > 0.05, two-sample *t*-test with Welch correction) in Na$_V$1.7 KO compared to WT dorsal horn neurons.

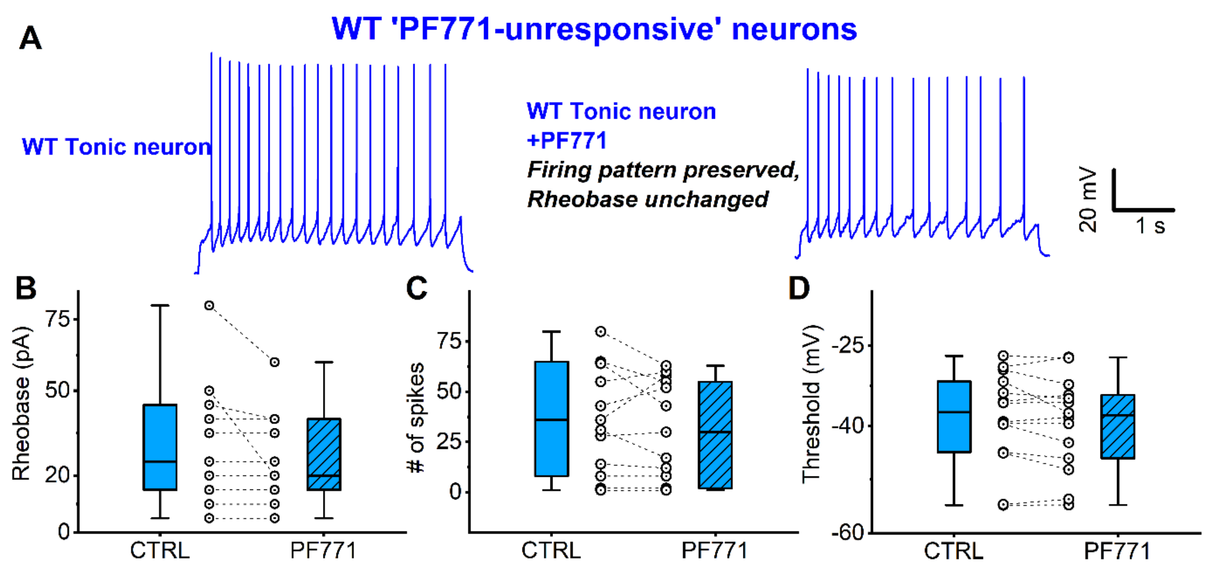

Fig. S4. "PF771-unresponsive" WT superficial dorsal horn neurons. Two populations of WT neurons were identified: neurons that displayed an increase in rheobase and neurons that showed little or no change in rheobase in the presence of PF771. (**A**) Representative WT tonic firing neuron displaying no effect of PF771 on firing pattern or rheobase. Current injection was 60 pA both before and after drug. There were no significant changes in (**B**) rheobase (**C**) # of spikes or (**D**) threshold of these neurons in the presence of PF771.
